# Author Correction: Proteolytic processing induces a conformational switch required for antibacterial toxin delivery

**DOI:** 10.1038/s41467-022-34174-z

**Published:** 2022-10-21

**Authors:** Nicholas L. Bartelli, Victor J. Passanisi, Karolina Michalska, Kiho Song, Dinh Q. Nhan, Hongjun Zhou, Bonnie J. Cuthbert, Lucy M. Stols, William H. Eschenfeldt, Nicholas G. Wilson, Jesse S. Basra, Ricardo Cortes, Zainab Noorsher, Youssef Gabraiel, Isaac Poonen-Honig, Elizabeth C. Seacord, Celia W. Goulding, David A. Low, Andrzej Joachimiak, Frederick W. Dahlquist, Christopher S. Hayes

**Affiliations:** 1 Department of Chemistry and Biochemistry, University of California, Santa Barbara, CA USA; 2 Midwest Center for Structural Genomics, Argonne National Laboratory, Lemont, IL USA; 3 Center for Structural Genomics of Infectious Diseases, University of Chicago, Chicago, IL USA; 4 Structural Biology Center, X-ray Science Division, Argonne National Laboratory, Lemont, IL USA; 5 Biomolecular Science and Engineering Program, University of California, Santa Barbara, CA USA; 6 Department of Molecular, Cellular and Developmental Biology, University of California, Santa Barbara, CA USA; 7 Department of Molecular Biology & Biochemistry, University of California, Irvine, CA USA; 8 Pharmaceutical Sciences, University of California, Irvine, CA USA; 9 Department of Biochemistry and Molecular Biology, University of Chicago, Chicago, IL USA

**Keywords:** Bacterial structural biology, Nucleases, Protein folding, Bacterial toxins

Correction to: *Nature Communications* 10.1038/s41467-022-32795-y, published online 29 August 2022

The original version of this Article contained an error in Table 1. The correct version of the 11th row of the 2nd column states ‘0.635’ instead of the original, incorrect ‘−0.635’. The correct version of the 12th row of the 2nd column states ‘0.881’ instead of the original, incorrect ‘−0.881’. This has been corrected in both the PDF and HTML versions of the Article.

