# Supplementary material for: Proteolytic processing induces a conformational switch required for antibacterial toxin delivery
Source: Nat Commun. 2022 Aug 29;13:5078. doi: 10.1038/s41467-022-32795-y (PMC9424206; doi:10.1038/s41467-022-32795-y)
Supplement: Supplementary file 1 — Supplementary Information [file 41467_2022_32795_MOESM1_ESM.pdf]

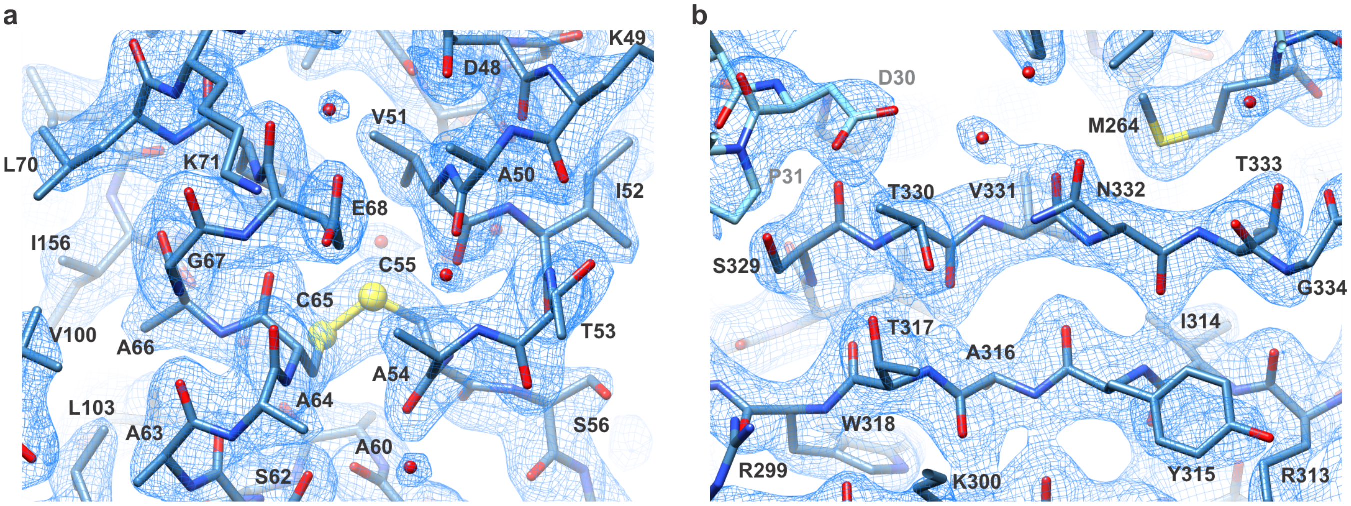

Supplementary Figure 1. Electron density maps. $2mF_o$-DF_c electron density maps contoured at 2σ level focusing on the disulfide bond in the CdiA-CT^{EC3006} entry domain (**a**), and the active site of the CdiA-CT^{EC3006} tRNase domain (**b**). The cyan side chains labeled in grey correspond to the CdiI^{EC3006} immunity protein. The structure dataset is available at the Protein Data Bank under accession 6VEK [http://doi.org/10.2210/pdb6VEK/pdb].

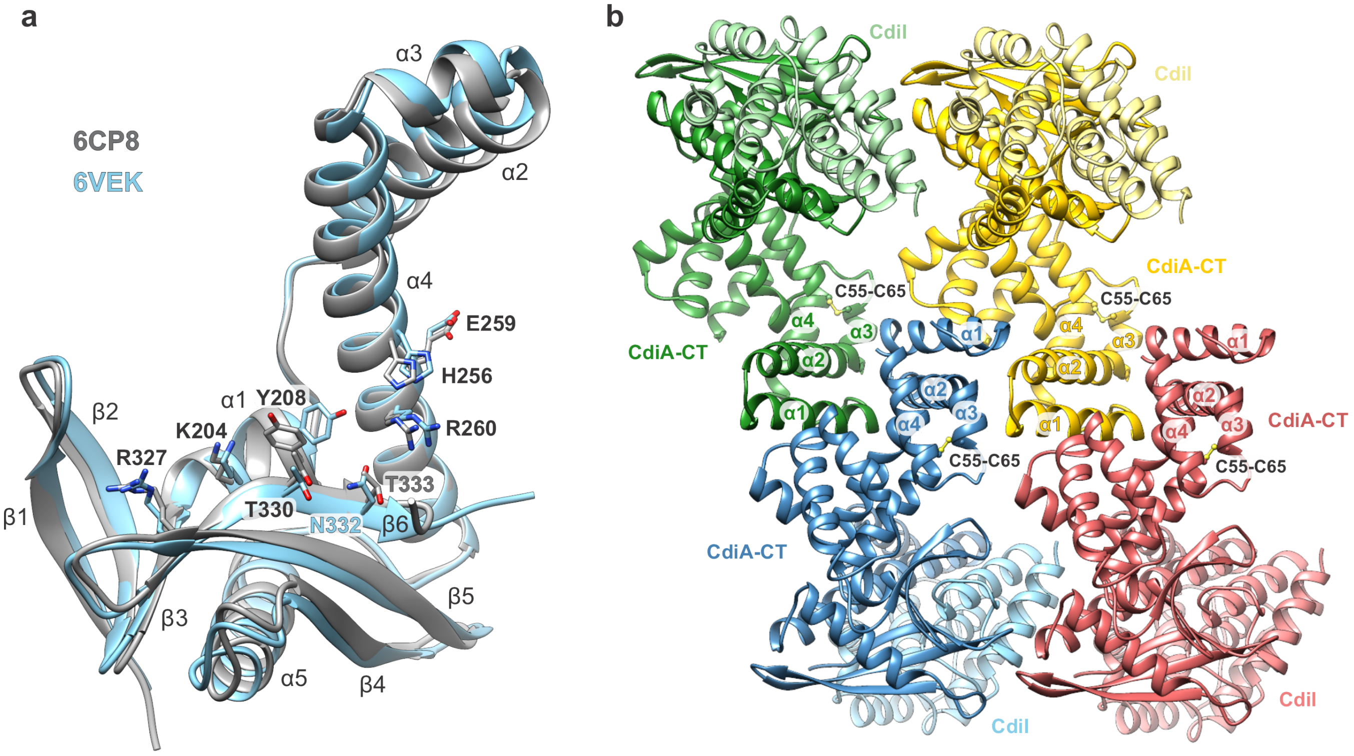

Supplementary Figure 2. Comparison of the tRNase domains between PDB:6VEK and 6CP8. a) The tRNase domain from PDB:6VEK [http://doi.org/10.2210/pdb6VEK/pdb] superimposes with rmsd of 0.94/0.91 Å over 150 Cα atoms of chains A and B present in the 6CP8 [http://doi.org/10.2210/pdb6CP8/pdb] structure. The absence of Asn332 in 6CP8 alters the trajectory of the C-terminal tail and results in rotation of active-site residue Tyr208. b) Crystal packing of the CdiA-CT•CdiI^{EC3006} complex in PDB:6VEK [http://doi.org/10.2210/pdb6VEK/pdb]. The Cys55-Cys65 disulfide linkage and secondary structure elements for the N-terminal subdomain of the cytoplasmic entry domain are indicated.

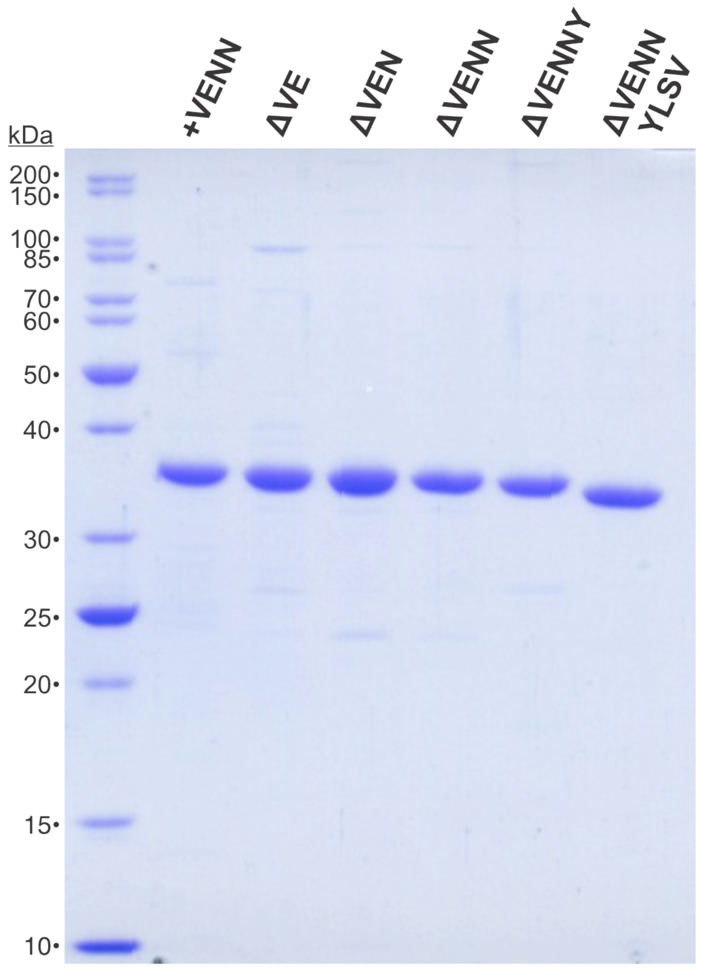

Supplementary Figure 3. SDS-PAGE of purified CdiA-CT^{EC3006} variants. Proteins were produced with N-terminal His_6 epitopes linked via tobacco etch virus (TEV) protease cleavage sites. Ni^{2+}-affinity purified proteins were digested with His_6-tagged TEV protease, then isolated for SDS-PAGE analysis. This experiment was performed once. Source data are presented as a Source Data file.

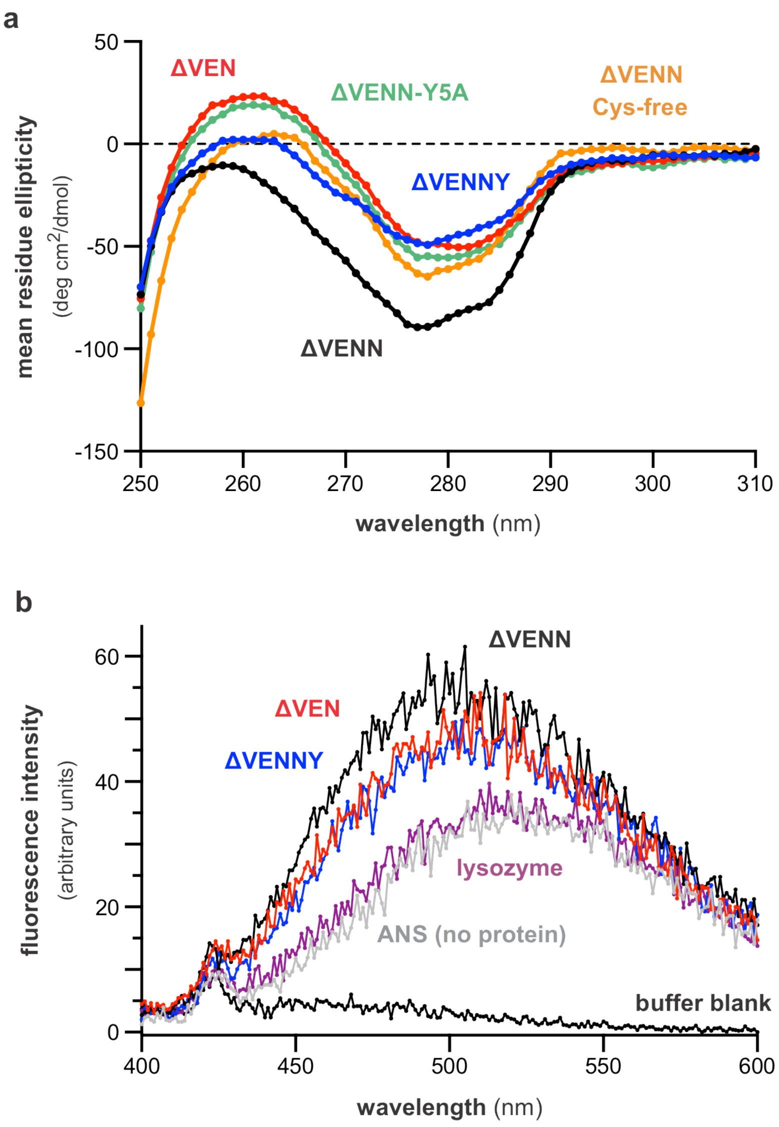

Supplementary Figure 4. Near UV CD spectroscopy and ANS fluorescence. a) CD spectra of PtsG-dependent entry domain variants. Presented data are the averages from three replicate scans. **b**) 8-anilino-1-naphthalenesulfonate (ANS) fluorescence. Purified proteins (6 µM final concentration) were incubated with 50 µM ANS and fluorescence emission monitored. Presented data are the averages from four technical replicates. The experiment in panel a was repeated independently twice with similar results. The experiment in panel b was performed once. Source data are presented as a Source Data file.

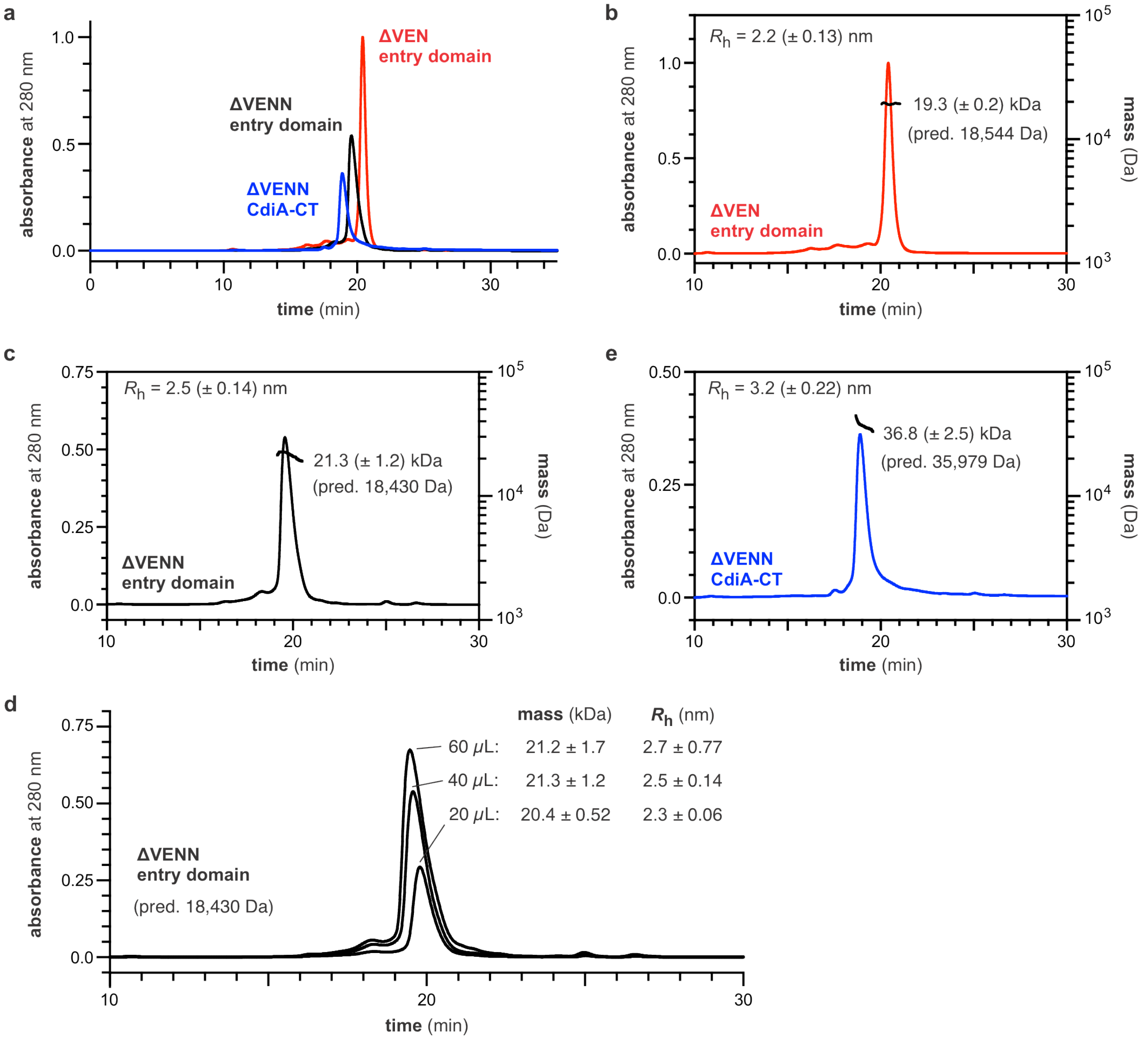

Supplementary Figure 5. Size-exclusion chromatography - multi-angle light scattering (SEC-MALS) analysis. a) Size-exclusion chromatography elution profiles. **b**) MALS analysis of the ΔVEN entry domain. Peak eluate concentration was 30 µM. **c**) MALS analysis of the ΔVENN entry domain. Peak eluate concentration was 16 µM. **d**) SEC-MALS analysis of the ΔVENN entry domain. The indicated volumes of purified ΔVENN entry domain were injected for analysis. Peak eluate concentrations were 9 µM (20 µL injection) and 21 µM (60 µL injection). **e**) MALS analysis of the ΔVENN CdiA-CT^{EC3006} construct. Molecular mass and hydrodynamic radius (R_h) measurements are presented as averages ± SD. All experiments were performed once. Source data are presented as a Source Data file.

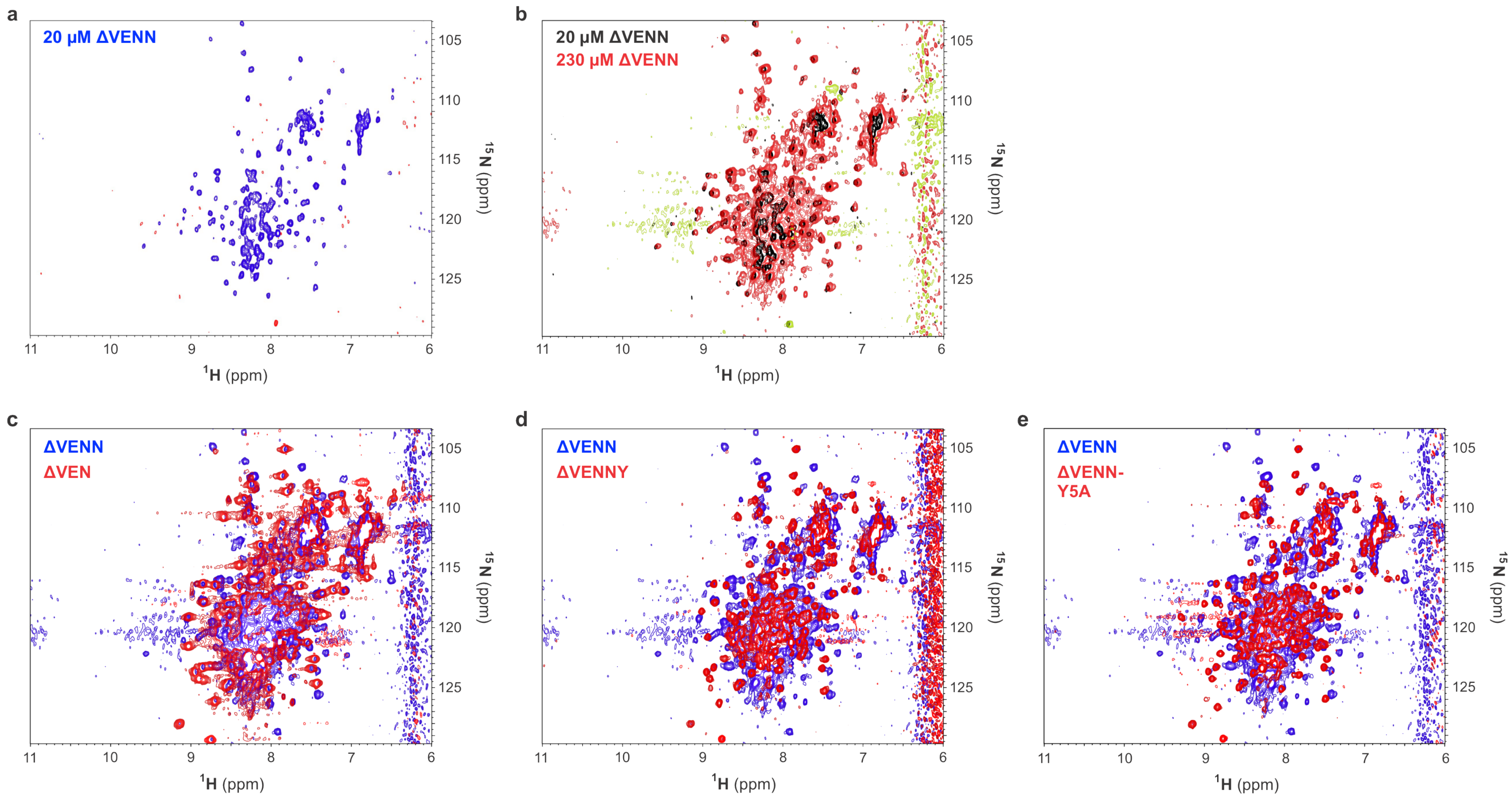

Supplementary Figure 6. Entry domain heterogeneity. **a**) ^{1}H-^{15}N HSQC spectrum of the ΔVENN entry domain at 20 μM. **b**) Overlay of 20 μM and 230 μM ΔVENN entry domain ^{1}H-^{15}N HSQC spectra at low-contour. **c**) Overlay of ΔVENN (230 μM) and ΔVEN (302 μM) entry domain ^{1}H-^{15}N HSQC spectra at low contour. **d**) Overlay of ΔVENN (230 μM) and ΔVENNY (135 μM) entry domain ^{1}H-^{15}N HSQC spectra at low contour. **e**) Overlay of ΔVENN (230 μM) and ΔVENN-Y5A (303 μM) entry domain ^{1}H-^{15}N HSQC spectra at low contour. All experiments were performed once. Source data are presented as a Source Data file.

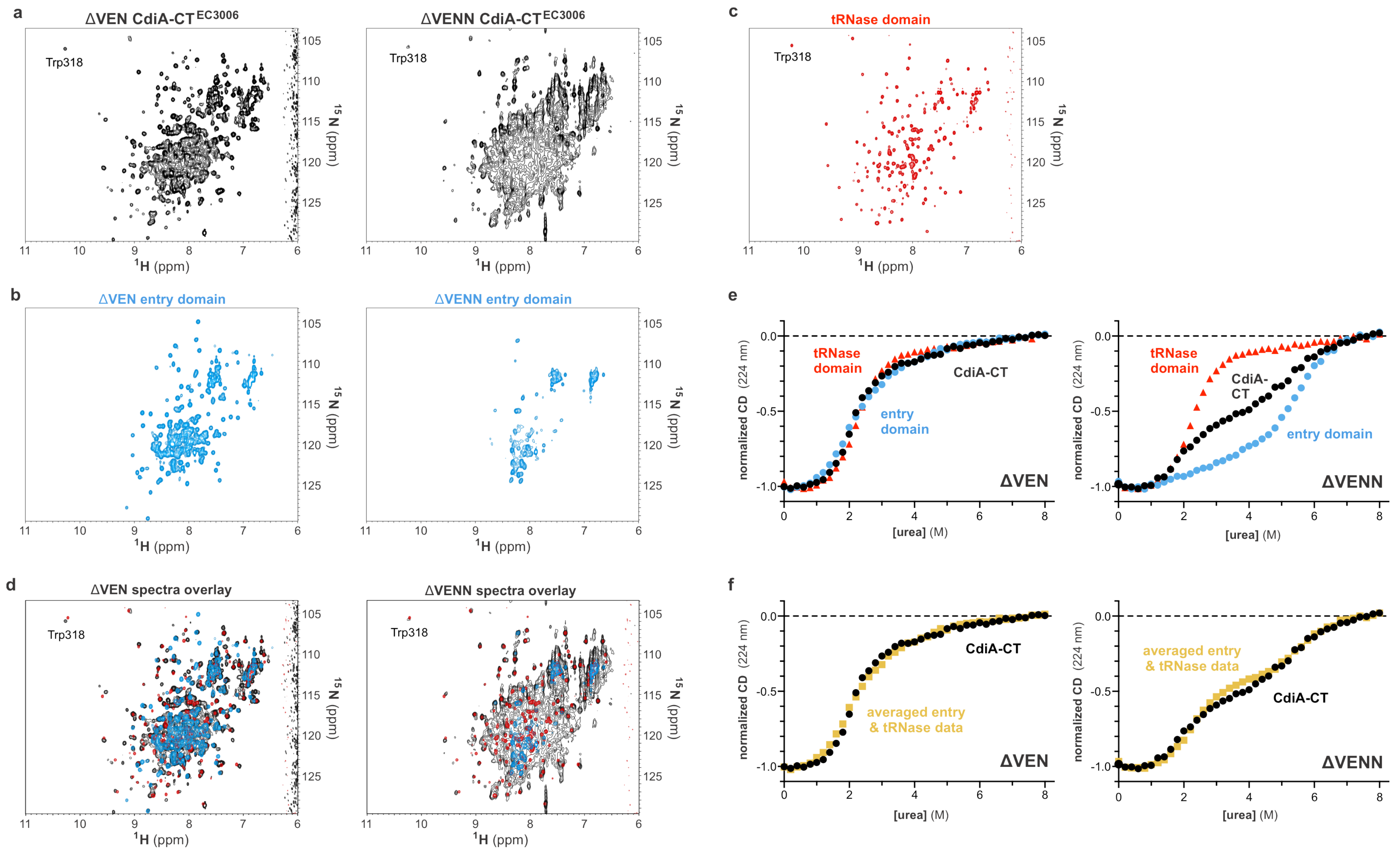

Supplementary Figure 7. Entry domain heterogeneity in the context of the intact CdiA-CT^{EC3006}. a) ^{1}H-^{15}N HSQC spectra of ΔVEN and ΔVENN versions of CdiA-CT^{EC3006}. **b**) ^{1}H-^{15}N HSQC spectra of ΔVEN and ΔVENN entry domains. **c**) ^{1}H-^{15}N HSQC spectrum of tRNase domain from CdiA-CT^{EC3006}. **d**) Overlays of entry domain, tRNase domain and CdiA-CT^{EC3006} spectra. **e**) Chemical denaturation profiles of the intact CdiA-CT^{EC3006} compared with isolated entry and tRNase domains. **f**) Comparison experimental CdiA-CT^{EC3006} denaturation to the averaged profiles for individual entry and tRNase domains. The experiments in panels a, b, c and d were performed once. Source data are presented as a Source Data file.

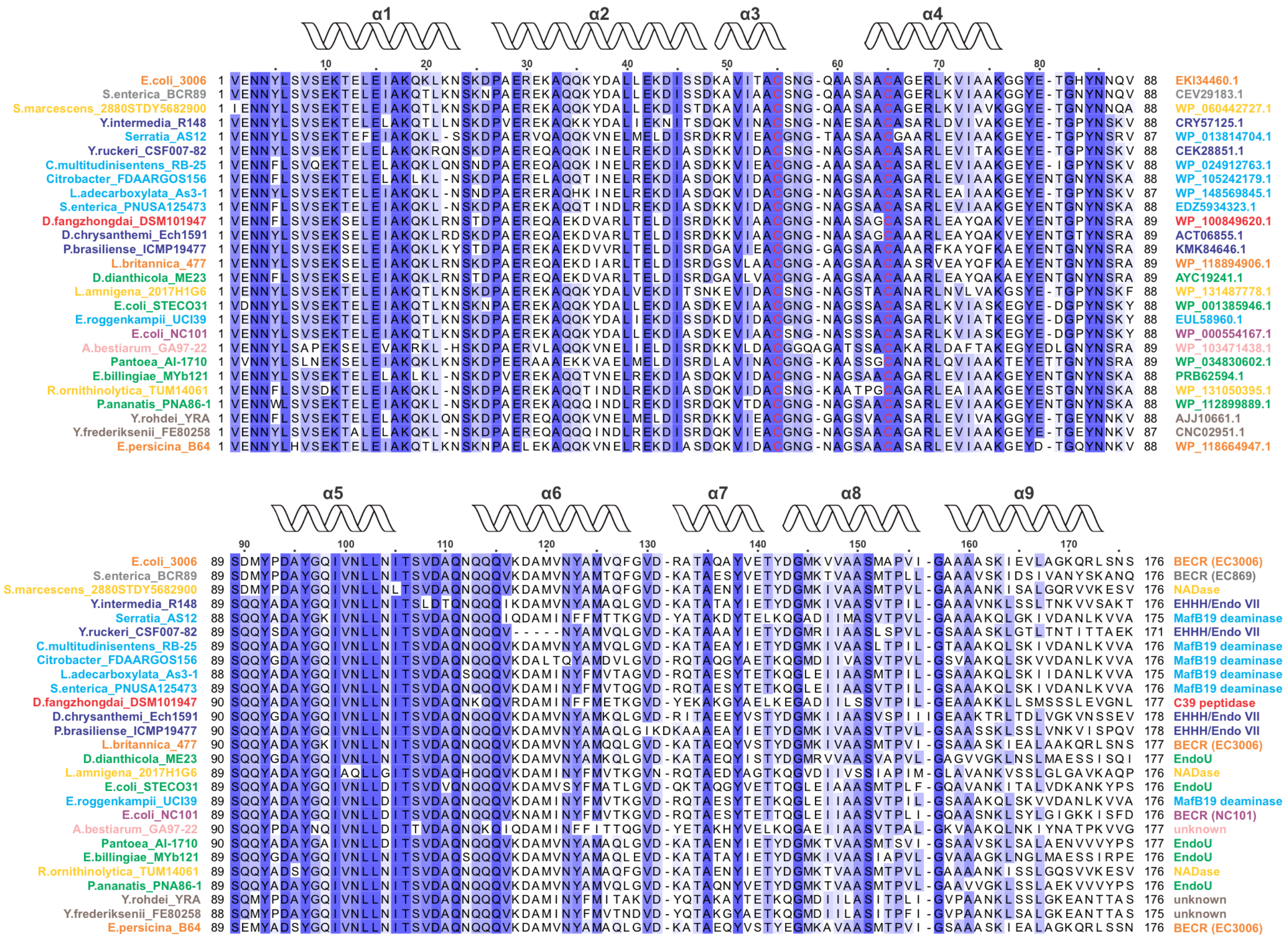

Supplementary Figure 8. Alignment of predicted PtsG-dependent entry domains. Secondary structure elements correspond those in the crystal structure of CdiA-CT^{EC3006}. Bacterial species and strains are indicated on the left, and NCBI reference numbers are provided to the right of the upper sequence block. All labels are color-coded according the identity of the C-terminal toxin domains, which are indicated to the right of the lower sequence block. Conserved Cys residues that form a disulfide in CdiA-CT^{EC3006} are rendered in red.

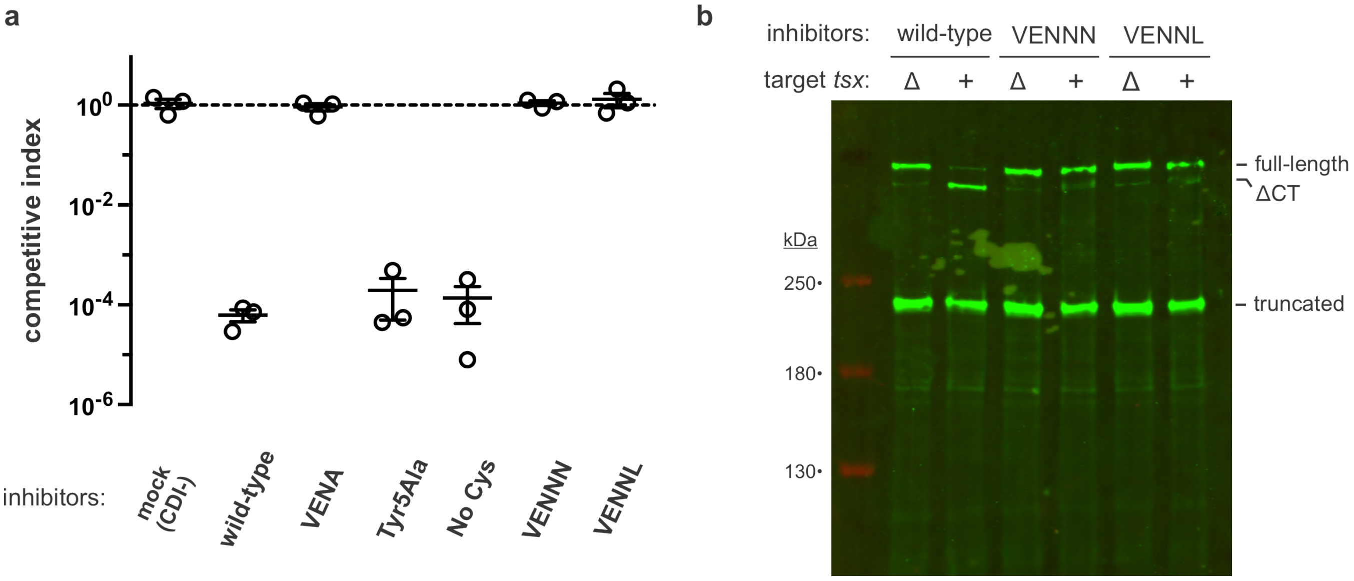

Supplementary Figure 9. Growth inhibition activities of CdiA-CT^{EC3006} variants. a) Inhibitor cell strains that deploy the indicated CdiA-CT^{EC3006} variants were mixed 1:1 with target bacteria in LB media and incubated for 1 h at 37 °C. Competitive indices equal the final ratio of viable target to inhibitor cells divided by the initial ratio. Data are presented as mean ± SEM for three independent experiments. **b**) Immunoblot analysis of chimeric CdiA. CdiA expressing inhibitor cells were mixed with *tsx*$^{+}$ or Δ*tsx* target cells. Proteins were analyzed by immunoblotting with antibodies to the N-terminal TPS domain of CdiA^{STECO31}. The migration positions of full-length, ΔCT and the main truncated form of CdiA are indicated on the right of the blot. The experiment in panel b was repeated independently twice with similar results. Source data are presented as a Source Data file.

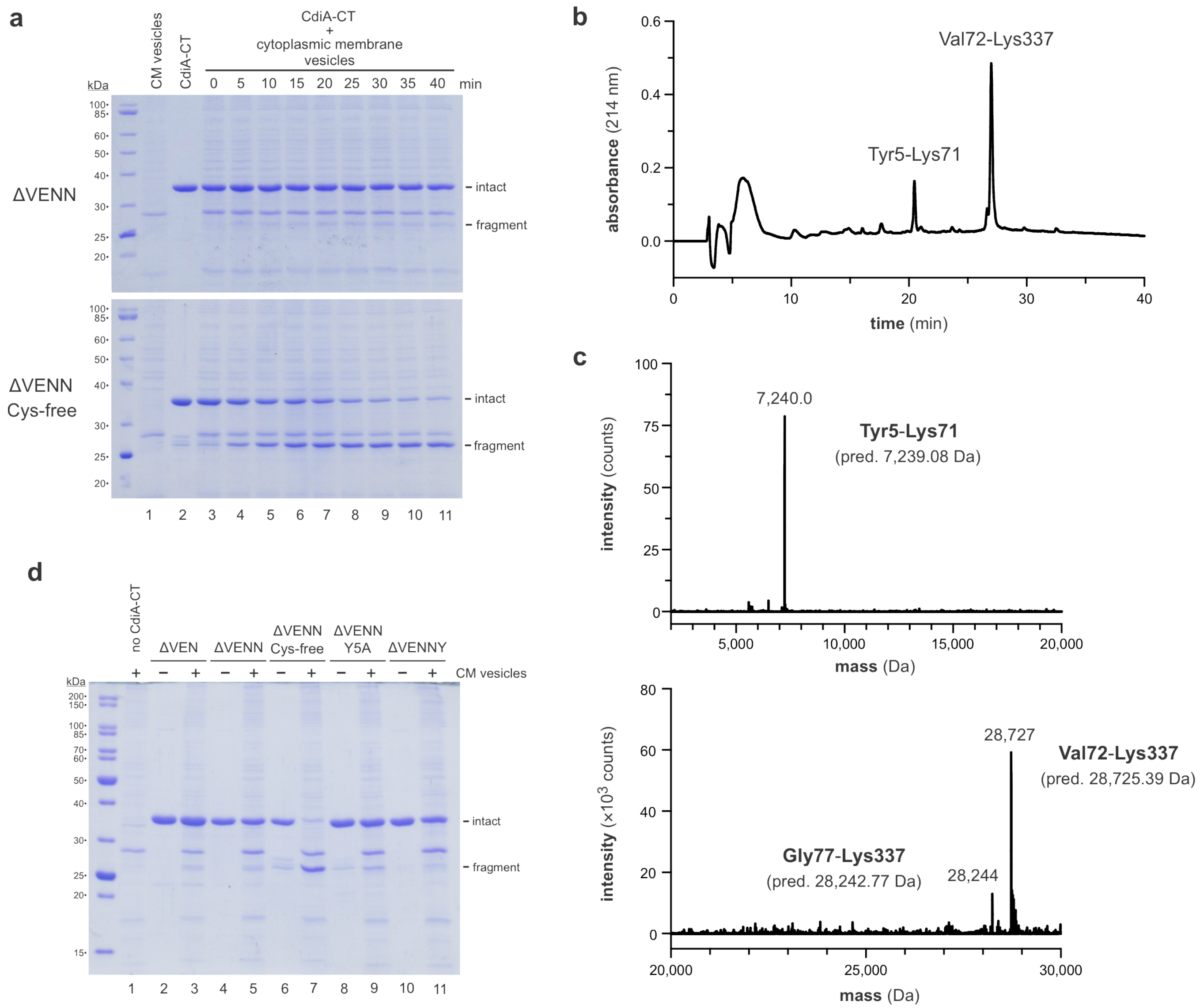

Supplementary Figure 10. The conserved disulfide protects CdiA-CT^{EC3006} from proteolysis. a) Purified wild-type and Cys-free versions of CdiA-CT^{EC3006} were incubated with cytoplasmic membrane vesicles in phosphate buffer (pH 5.5) at ambient temperature and analyzed by SDS-PAGE. **b**) Reverse phase-HPLC analysis of Cys-free CdiA-CT^{EC3006} digest. **c**) Electrospray ionization-mass spectrometry of HPLC resolved peptide fragments from panel **b**. Identified fragments and their predicted masses are indicated. **d**) CdiA-CT^{EC3006} variants were incubated with or without cytoplasmic membrane vesicles for 40 min and analyzed by SDS-PAGE. The experiment in panel a was repeated independently twice with similar results. The experiments from panels b, c and d were performed once. Source data are presented as a Source Data file.

Supplementary Table 1. Enterobacterial cytoplasm entry domain families.

family	receptor	associated toxin domains	toxin/homolog structures PDB ID	CdiA effector	species and strain
**1**	MetI	Endonuclea_NS_2 (PF13930)	2XGR; 2XH3	WP_097339041.1	*Escherichia coli* MOD1-EC5140
		unknown	5T86	WP_001081264.1	*Escherichia coli* EC1738
		DUF4258 (PF14076)	2FHZ	WP_056235768.1	*Erwinia* sp. Leaf53
		RNase T1 (PF00545)	1PYL; 1MGR	WP_014915291.1	*Pectobacterium carotovorum* subsp. carotovorum PCC21
		colicin E3 (PF09000)	1E44	WP_011093679.1	*Pectobacterium atrosepticum* SCRI1043
**2**	PtsG	Ile tRNase (cd20723)	6CP9; 6VEK	EKI34460.1	*Escherichia coli* 3006
		Gln/Asn tRNase		CEV29183.1	*Salmonella enterica* subsp. enterica serovar Typhi MDUST41
		NADase (PF14021)	4QLP; 6B12	WP_016151891.1	*Citrobacter* sp. KTE151
		EndoU (PF14436)	5HKQ	WP_001385946.1	*Escherichia coli* STEC_O31
		BECR toxin	7M5F	AJJ10661.1	*Yersinia rohdei* YRA
		Ntox48 (no Pfam)		CEK28851.1	*Yersinia ruckeri* CSF007-82
		MafB19-deam (PF14437)	1ALN; 1R5T	WP_084832595.1	*Enterobacter roggenkampii* UCI 39
		Glu/Asp tRNase	5I4Q; 5I4R	WP_000554167.1	*Escherichia coli* NC101
		Peptidase_C39 (PF03412)	3K8U	WP_100849620.1	*Dickeya fangzhongdai* DSM 101947
**3**	FtsH	Ntox28 (PF15605)	5J43; 5J5V	WP_000554175.1	*Escherichia coli* 536
		Ntox21 (PF15526)	4NTQ	WP_013098820.1	*Enterobacter cloacae* ATCC 13047
		BECR tRNase	6D7Y	WP_052135798.1	*Raoultella planticola* FDAARGOS_64
		unknown		WP_081251380.1	*Dickeya solani* IPO 2222
		unknown		CFR26299.1	*Yersinia frederiksenii* FCF467
		unknown	5T87	WP_076945893.1	*Raoultella terrigena* NZ133
		BECR RNase (COG5529)		AHK21648.1	*Yersinia similis* 228
**4**	YciB	DNase (cd13444)	4G6U; 4ZQU	EGI36612.1	*Escherichia coli* TA271
		toxin-ParB (cd16392)	2HWJ	OKP23932.1	*Serratia liquefaciens* 72
		BECR toxin	7M5F	WP_060446815.1	*Serratia marcescens* BWH57
**5**	RbsC	putative NADase	3PNT	ADM98580.1	*Dickeya dadantii* 3937
		unknown		HAK1938778.1	*Salmonella enterica* MA.R291
		unknown		HAU5566446.1	*Serratia fonticola*
		unknown		WP_103795907.1	*Pantoea* sp. PSNIH6
		BpE479 tRNase (PF18664)	5J4A	MRT42876.1	Enterobacteriaceae bacterium RIT702
**6**	GltJK	LHH/Endo VII (PF14411)	6W0V; 4UHP	CAE12843.1	*Photorhabdus luminescens* subsp. laumondii TTO1
		endonuclease (?)	5GKE	WP_023045880.1	*Photorhabdus temperata* J3
		endonuclease (?)	5GKE; 2VLD	KMW73064.1	*Photorhabdus luminescens* subsp. luminescens DSM 3368
**7***	SecY	pore-forming		WP_001081254.1	*Escherichia coli* B799
		MafB19-deam (PF14437)	1ALN; 1R5T	WP_006660219.1	*Providencia alcalifaciens* DSM 30120
		EndoU (PF14436)	5HKQ	WP_144421361.1	*Cronobacter dublinensis* LMG 23823
		Bp1026b tRNase (cd13442)	4G6V	APC10535.1	*Providencia rettgeri* RB151
**8***	AcrB	EndoU (PF14436)	5HKQ	WP_004876812.1	*Yersinia mollaretii* ATCC 43969
		RNase T1 (PF00545)	1GOU; 2C4B	WP_020439873.1	*Serratia plymuthica* S13
**9***	SbmA	BECR RNase (COG5529)		EIF16908.1	*Escherichia coli* O32:H37 str. P4
		Ntox50 (PF15542)		WP_110534649.1	*Klebsiella pneumoniae* BIDMC 52
		Tox-HNH-EHHH (PF15657)		WP_080314656.1	*Enterobacter cloaca*e GN6
		PD-(D/E)*x*K		SQZ92406.1	*Escherichia coli* 3-267-03_S3_C2
		Glu/Asp tRNase	5I4Q; 5I4R	WP_064735329.1	*Klebsiella pneumoniae* MGH 68
		BECR tRNase	6D7Y	WP_080397706.1	*Enterobacter cloacae* subsp. cloacae SMART_886
		DUF4258 (PF14076)	2FHZ	WP_108416527.1	*Enterobacter mori* WCHEM045008
		unknown		ESN44960.1	*Klebsiella variicola* MGH 20
		unknown		ESN17358.1	*Enterobacter* sp. MGH 24
**10**	MurP	Toxin-deaminase (PF14424)	5K83; 2M65	WP_005186610.1	*Yersinia intermedia* ATCC 29909

		Bp1026b tRNase (cd13442)	4G6V	WP_037397371.1	*Serratia* sp. Ag1
		BpE479 tRNase (PF18664)	5J4A	WP_051916762.1	*Serratia* sp. Ag1
		DNase_NucA_NucB (PF14040)	5OMT	WP_099137702.1	*Xenorhabdus innexi* DSM 16336
**11**	unknown	BECR tRNase	6D7Y	EIG93024.1	*Escherichia coli* 97.0246
		DUF769 (PF05590)	1SGV	WP_001081262.1	*Escherichia coli* DEC9E
		DUF4258 (PF14076)		WP_096965290.1	*Escherichia coli* MOD1-EC6823
**12**	unknown	Endonuclea_NS_2 (PF13930)	2XGR; 2XH3	WP_079893968.1	*Salmonella enterica*
		Gln/Asn tRNase		ADO09110.1	*Pantoea vagans* C9-1
		cytidine deaminase (?)	1MQ0; 2D30	WP_050568187.1	*Cronobacter muytjensii* ATCC 51329
		Bp1026b tRNase (cd13442)	4G6V	WP_012147097.1	*Serratia proteamaculans* 568
**13**	unknown	Gln/Asn tRNase		WP_001075571.1	*Escherichia coli* EC869
		EndoU (PF14436)	5HKQ	WP_163843187.1	*Pantoea agglomerans* T6
		BECR (?)	6CP9; 6CP8	WP_081049483.1	*Pantoea stewartii* RSA30
		Ntox19 (no Pfam)		WP_052953278.1	*Enterobacter cloacae* CH1
		unknown		WP_052447813.1	*Serratia symbiotica* CWBI-2.3
		NADase (?)	6B12; 4QLP	PXW48285.1	*Erwinia* sp. AG740
		BpE479 tRNase (PF18664)	5J4A	AHY09250.1	*Serratia plymuthica* V4
		NADase (PF14021)	4QLP; 6B12	WP_080725787.1	*Yersinia kristensenii* ATCC 33641
**14**	unknown	NADase (PF14021)	4QLP	WP_029488456.1	*Escherichia coli* FCH1
		DUF769 (PF05590)		WP_062745413.1	*Erwinia persicina* NBRC 102418
		EndoU (PF14436)	5HKQ	WP_155960930.1	*Klebsiella aerogenes* GN06193
		NADase (?)	6B12: 4QLP	SHG12251.1	*Pectobacterium carotovorum* DSM 30168
		unknown		WP_052696968.1	*Pantoea* sp. SM3
**15**	unknown	RNase_A_bac (PF18431)	5E3E	EEP92680.1	*Yersinia kristensenii* ATCC 33638
		colicin E3 (PF09000)	1E44	CNL08982.1	*Yersinia frederiksenii* RS-42
		Ile tRNase (cd20723)	6CP9; 6VEK	WP_012104437.1	*Yersinia pseudotuberculosis* IP 31758
		Bp1026b tRNase (cd13442)	4G6V	WP_022622695.1	*Pantoea ananatis* BRT175
		BpE479 tRNase (PF18664)	5J4A	WP_071925993.1	*Serratia fonticola* GS2
		unknown		CNG94455.1	*Yersinia frederiksenii* IP23698
		colicin D (PF11429)	1V74; 1TFK	CFQ98836.1	*Yersinia frederiksenii* 3400/83
		unknown		CQJ05208.1	*Yersinia frederiksenii* RS-42
		Gln/Asn tRNase		KGA44894.1	*Yersinia frederiksenii* ATCC 33641
		DUF769 (PF05590)		WP_039294138.1	*Cedecea neteri* M006
		Ntox17 (PF15524)		KGB00281.1	Enterobacteriaceae bacterium ATCC 29904
		Ntox19 (no Pfam)		WP_085687401.1	*Lonsdalea populi* N-5-1
		Ntox7 (no Pfam/BECR)		CAR67723.1	*Photorhabdus asymbiotica* subsp. asymbiotica ATCC 43949
**16**	unknown	DNase		E0SDG8.1	*Dickeya dadantii* 3937
		Endonuclea_NS_2 (PF13930)	2XGR; 2XH3	WP_058775902.1	*Pantoea dispersa* SA2
		DUF4258 (PF14076)	2FHZ	WP_085286274.1	*Serratia marcescens* MGH223
		PD-(D/E)*x*K		CNL05979.1	*Yersinia aldovae* IP06005
		unknown		CNH44427.1	*Yersinia massiliensis* 24070
		LHH/Endo VII (PF14411)	4UHP; 6W0V	WP_005188854.1	*Yersinia intermedia* ATCC 29909
		colicin D (PF11429)	1V74; 5ZNM	AFR03661.1	*Pectobacterium carotovorum* subsp. carotovorum PCC21
		Ntox7 (no Pfam/BECR)		WP_051880442.1	*Buttiauxella noackiae* MCE
		endonuclease (?)	1M0D	WP_098938818.1	*Pluralibacter gergoviae* FDAARGOS_386
**17***	unknown	Ntox19 (no Pfam)		GAE11957.1	Yersinia pseudotuberculosis NBRC 105692
		unknown		ACA67915.1	*Yersinia pseudotuberculosis* YPIII
		Peptidase_C70 (PF12385)	6HQZ	WP_096864820.1	*Providencia rettgeri* BML2496
**18**	unknown	colicin E3 (PF09000)	1E44	AAN38708.1	*Dickeya chrysanthemi* EC16
		unknown	5T86	EIQ74285.1	*Shigella flexneri* 1235-66
		unknown	5T87	CAR66715.1	*Photorhabdus asymbiotica* subsp. asymbiotica ATCC 43949
		ParB-like (?)	6KY4; 5UJD	WP_036152621.1	*Lonsdalea quercina* subsp. quercina ATCC 29281

**19**	unknown	unknown		WP_021016158.1	*Serratia* sp. ATCC 39006
		Ntox19 (no Pfam)		CNG87290.1	*Yersinia enterocolitica* ERL08708
		PD-(D/E)xK (cd22341)	2VLD	WP_115586245.1	*Cedecea davisae* DSM 4568
**20**	unknown	unknown		ACX86275.1	*Pectobacterium parmentieri* WPP163
		unknown		CNK27099.1	*Yersinia enterocolitica* YE8850
		DNase (cd13444)	4G6U; 4ZQU	WP_024557922.1	*Franconibacter pulveris* 1160
		Endonuclea_NS_2 (PF13930)	2XGR; 2XH3	WP_013185149.1	*Xenorhabdus nematophila* ATCC 19061
**21**	unknown	unknown		WP_014915690.1	*Pectobacterium carotovorum* subsp. carotovorum PCC21
		unknown		WP_095699665.1	*Pectobacterium polaris* NIBIO1392
		Endonuclea_NS_2 (PF13930)	2XGR; 2XH3	WP_006657883.1	*Providencia alcalifaciens* DSM 30120
**22**	unknown	unknown		WP_083239551.1	*Enterobacter* sp. HK169
		ParB-like nuclease	1VZ0	WP_081273791.1	*Pantoea agglomerans* pv. betae 4188
		Ntox21 (PF15526)	4NTQ	WP_200925706.1	*Enterobacter roggenkampii* STW0522-66
		BECR (?)	6CP9; 6CP8	WP_052720638.1	*Pantoea* sp. 3.5.1
		unknown	5T87	WP_015697204.1	*Rahnella aquatilis* CIP 78.65
**23**	unknown	MafB19-deam (PF14437)	1WKQ; 1ALN	WP_080774704.1	*Yersinia intermedia* Y228
		unknown		WP_033733079.1	*Pantoea vagans* MP7
		Bp1026b tRNase (cd13442)	4G6V	WP_024913292.1	*Chania multitudinisentens* RB-25
		BpE479 tRNase (PF18664)	5J4A	WP_038916980.1	*Dickeya zeae* NCPPB 2538
		unknown		WP_094959878.1	*Proteus mirabilis* PM187
		cytidine deaminase (?)	3B8F	WP_036976965.1	*Proteus mirabilis* ATCC 7002
		EndoU (PF14436)	5HKQ	WP_098943415.1	*Proteus vulgaris* FDAARGOS_366
**24**	unknown	RNase T1 (PF00545)	1PYL; 1MGR	WP_080984355.1	*Yersinia similis* MW109-2
		unknown		CRY71034.1	*Yersinia pseudotuberculosis* SP93422
		unknown	5T87	CQR16419.1	*Yersinia mollaretii* 64/02
		unknown		WP_099125154.1	*Xenorhabdus stockiae* DSM 17904
		RNase_A_bac (PF18431)	5E3E	OCA55965.1	*Photorhabdus luminescens* PB45.5
		cytidine deaminase (?)	1MQ0	WP_088373629.1	*Photorhabdus luminescens* HIM3
		unknown		AKH64116.1	*Photorhabdus thracensis* DSM 15199
		EndoU (PF14436)	5HKQ	WP_094419452.1	*Kosakonia cowanii* Esp_Z
		unknown		WP_083699370.1	*Kosakonia cowanii* 888-76
		unknown		WP_024562278.1	*Franconibacter helveticus*1159
**25**	unknown	NADase (PF14021)	4QLP; 6B12	WP_080479046.1	*Serratia marcescens* 945174350
		Ntox7 (no Pfam/BECR)		WP_033634711.1	*Serratia marcescens* BIDMC 44
		Ntox17 (PF15524)		WP_111533677.1	*Pantoea agglomerans* BI3
		EndoU (PF14436)	5HKQ	WP_080987661.1	*Yersinia pseudotuberculosis* IP32670
		DUF769 (PF05590)		WP_064740235.1	*Enterobacter ludwigii* EnVs2
		Ntox50 (PF15542/BECR)		WP_080774681.1	*Yersinia intermedia* Y228
		Gln deamidase (PF15644)	3B21	WP_080350532.1	*Enterobacter cloacae* 1161_ECLO
		unknown		EFE97198.1	*Serratia odorifera* DSM 4582
		NADase (?)	6B12; 6YGG	WP_038482520.1	*Cedecea neteri* SSMD04
		unknown		WP_101077391.1	*Rahnella* sp. AA
		methyltransferase (?)	1EG2	WP_023478991.1	*Enterobacter cloacae* S611
**26**	unknown	BECR RNase (COG5529)		SET02244.1	*Enterobacter* sp. NFIX09
		Ntox28 (PF15605)	5J43; 5J5V	WP_128297714.1	*Enterobacter cloacae* GEO_30_Eff_A
		BECR tRNase	6D7Y	WP_110268579.1	*Pantoea* sp. PNA 03-3
		Ntox33 (PF15533)		WP_083451851.1	*Photorhabdus heterorhabditis* VMG
**27**	unknown	unknown		WP_105720178.1	*Cronobacter dublinensis* cro3540W
		unknown		WP_161591729.1	*Cronobacter dublinensis* SX10
		EndoU (PF14436)	5HKQ	WP_108901425.1	*Limnobaculum parvum* HYN0051
		unknown		WP_053007602.1	*Pragia fontium* 24613
**28**	unknown	Ntox33 (PF15533)		WP_080725786.1	*Yersinia frederiksenii* ATCC 33641

		Ntox21 (PF15526)	4NTQ	WP_098904966.1	*Yersinia frederiksenii* FDAARGOS_417

**29**	unknown	RNase T1 (PF00545)	1PYL; 1MGR	WP_080991559.1	*Yersinia intermedia* 58735
		unknown		CNC78714.1	*Yersinia frederiksenii* FE80151
		EndoU (PF14436)	5HKQ	WP_105517296.1	*Cronobacter sakazakii* MOD1_Jor44
		colicin D (PF11429)	1V74; 1TFK	WP_071430009.1	*Tatumella* sp. TA1
		Gln/Asn tRNase		WP_046288390.1	*Pantoea* sp. 3.5.1

*****Entry domain families that lack paired Cys residues

Supplementary Table 2. Bacterial strains.

Strain	Description[a]	Reference of source
BL21(DE3)	*fhuA2 [lon] ompT gal (λ DE3) [dcm] ΔhsdS*	Agilent
DH5α	*endA1 hsdR17 glnV44 thi-1 recA1 gyrA96 relA1φ80dlacZΔM15 Δ(lacZYA-argF)U169*, Str^R	Stratagene
DH5α pir^+	*endA1 hsdR17 glnV44 thi-1 recA1 gyrA96 relA1φ80dlacZΔM15 Δ(lacZYA-argF)U169 zdg-232::Tn10 uidA::*pir^+, Tet^R Str^R	Stratagene
MG1655	wild-type *E. coli* K-12 strain	lab collection
EPI100	*Δ(ara, leu)7697 galU galK* $λ^-$ *rpsL nupG*, Str^R	Epicentre
X90	F´ *lacI*^{q} *lac´ pro´/ara Δ(lac-pro) nal1 argE(amb) rif*^{r} *thi-1*, Rif^R	lab collection
CH1672	MG1655 Δ*wzb* Δ*tsx* Δ*ptsG*	this study
CH1673	MG1655 Δ*wzb* Δ*tsx* Δ*acrB*	this study
CH1738	MG1655 Δ*wzb* Δ*acrB::kan,* Kan^R	this study
CH1739	MG1655 Δ*wzb* Δ*acrB*	this study
CH2016	X90 (DE3) Δ*rna* Δ*slyD::kan,* Rif^R Kan^R	[60]
CH7157	X90 Δ*clpX* Δ*clpA::kan,* Rif^R Kan^R	[12]
CH7286	MG1655 Δ*wzb::kan,* Kan^R	[2]
CH7367	MG1655 Δ*wzb*	[2]
CH12743	MG1655 Δ*ptsG::kan,* Kan^R	this study
CH13258	X90 Δ*acrB::kan,* Rif^R Kan^R	this study
CH14016	MG1655 Δ*wzb* Δ*tsx*	[2]
DL8705	MG1655 Δ*wzb ara::spc,* $Spec^R$	[22]

[a]Abbreviations: Kan^R, kanamycin-resistant; Rif^R, rifampicin-resistant; $Spec^R$, spectinomycin-resistant; Str^R, streptomycin-resistant; Tet^R, tetracycline-resistant

Supplementary Table 3. Plasmids.

Plasmid	Description[a]	Reference or source
pCH368	pMCSG63::EC3006-ΔVENN-(Y5A)-cdiI, Amp^R	this study
pCH391	pMCSG63::EC3006-ΔVEN-CED, Amp^R	this study
pCH392	pMCSG63::EC3006-ΔVENN-CED, Amp^R	this study
pCH393	pMCSG63::EC3006-ΔVENN-(C55S-C65S)-CED, Amp^R	this study
pCH394	pMCSG63::EC3006-ΔVENN-(Y5A)-CED, Amp^R	this study
pCH396	pMCSG63::EC3006-ΔVENNY-CED, Amp^R	this study
pCH465	pMCSG63::Ymol43969-ΔVENN-cdiI, Amp^R	this study
pCH523	pET21b::STEC3-cdiBA-EC3006-CT(VENNN)-cdiI, Amp^R	this study
pCH524	pET21b::STEC3-cdiBA-EC3006-CT(VENNL)-cdiI, Amp^R	this study
pCH789	pMCSG63::EC3006-tRNase(R260A)-cdiI, Amp^R	this study
pCH891	pMCSG63::Yi29909-ΔVENN-CED, Amp^R	this study
pCH978	pMCSG58::EC3006-CT-cdiI, Amp^R	[25]
pCH1188	pMCSG63::Yi29909-ΔVEN-CED, Amp^R	this study
pCH1681	pET21b::STEC3-cdiBA-Ym43969-CT-cdiI, Amp^R	this study
pCH1735	pCH450(DAS)::Ym43969-CT-cdiI, Tet^R	this study
pCH1737	pTrc99aKX::Ym43969-CT-cdiI, Amp^R	[26]
pCH1741	pZS21-acrB, Kan^R	[67]
pCH5019	pET21b::STEC3-cdiBA-EC3006-CT-cdiI, Amp^R	this study
pCH6283	pUC57::EC3006-CT-cdiI, Amp^R	Genscript and [25]
pCH6587	pET21b::STEC3-cdiBA-EC3006-CT(N4A)-cdiI, Amp^R	this study
pCH6588	pET21b::STEC3-cdiBA-EC3006-CT(Y5A)-cdiI, Amp^R	this study
pCH6590	pET21b::STEC3-cdiBA-EC3006-CT(C55S-C65S)-cdiI, Amp^R	this study
pCH7171	pCH450(DAS)::EC536-CT-cdiI, Tet^R	[45]
pCH11526	pTrc99aKX::EC3006-cdiI, Amp^R	[18]
pCH11840	pTrc99a::ptsG, Amp^R	[18]
pCH12802	pET21::EC3006-cdiI, Amp^R	[18]
pCH12847	pDAL879::EC93-cdiBA-Ym43969-CT-cdiI, Cm^R	[26]
pCH13658	pET21b::STEC3-cdiBAI(no MCS), Amp^R	[2]
pCH13709	pET21b::STEC3-cdiBA-ΔCT/ΔcdiI, Amp^R	this study
pCH14272	pMCSG63::EC3006-ΔVENN-cdiI, Amp^R	this study
pCH14273	pMCSG63::EC3006-ΔVEN-cdiI, Amp^R	this study
pCH14274	pMCSG63::EC3006-ΔVE-cdiI, Amp^R	this study
pCH14275	pMCSG63::EC3006-ΔVENN-(C55S-C65S)-cdiI, Amp^R	this study
pCH14283	pMCSG63::EC3006-ΔVENNY-cdiI, Amp^R	this study
pCH14284	pMCSG63::EC3006-ΔVENNYSLV-cdiI, Amp^R	this study
pCP20	expresses FLP recombinase under heat-shock control, Cm^R Amp^R	[47]
pET21b	phage T7 RNA polymerase based expression plasmid, Amp^R	Novagen
pMCSG63	phage T7 RNA polymerase based expression plasmid, Amp^R	[49]
pMCSG58	phage T7 RNA polymerase based expression plasmid, Amp^R	[49]
pMCSG63-200209	pMCSG63::EC3006-CT-cdiI, Amp^R	[25]
pMCSG58-200209	pMCSG58::EC3006-CT-cdiI, Amp^R	this study
pSC189	mating mariner plasmid, Kan^R Amp^R	[64]
pTrc99A	IPTG-inducible expression plasmid, Amp^R	Amersham/Pharmacia
pZS21	pSC101-derivative, Kan^R	[66]

[a]Abbreviations: Amp^R, ampicillin-resistant; Cm^R, chloramphenicol-resistant; Kan^R, kanamycin-resistant; Tet^R, tetracycline-resistant

Supplementary Table 4. Oligonucleotides.

Identifier	Descriptor	Sequence[a]
209F46		5´ - GTC TCT CCC ATG GTT GAG AAT AAT TAT CTT AGC GTG TCT GAA AAG A
209R50		5´ - TGG TGG TGC CCA GCA TTA TTC AGA GGA TAA GCT TTT GAA AAA TCA TCG TA
CH577	Ile1 probe	5´ - ACC GAC CTC ACC CTT ATC AG
CH1417	tRNA-Glu probe	5´ - CCC CTG TTA CCG CCG TG
CH2260	mariner-rev-seq	5´ - AGG TCC AAT TCT CGT TTT CAT ACC TCG
CH3245	EC3006-cdiI-Xho-rev	5´ - GAC TCG AGT TAA TTA TTC AGA GGA TAA GC
CH3871	EC3006-C55A/C65A-rev	5´ - TCA CCG GCA CTA GCT GCG CTG GCT GCC TGT CCG TTA CTG CTC GCA GTG ATT AC
CH3975	YmoI-CT-Kpn/Nco-for	5´ - TTT GGT ACC ATG GTT GAG GAT AAT AAC CTT AG
CH3977	YmoI-cdiI-Spe-rev	5´ - TTT ACT AGT AGC AGG TAA TTT AGT CAG
CH4262	EC93-A2894-Nhe-for	5´ - AAA GCT AGC GCC GGT ACG GGG GC
CH4280	EC3006-C55A/C65A-for	5´ - GTA ATC ACT GCG AGC AGT AAC GGA CAG GCA GCC AGC GCA GCT AGT GCC GGT GA
CH4282	STEC-Afl/Sph-for	5´ - TTT GCA TGC TTA AGG CCG GTG GTA ACA C
CH4283	STEC-S2917-Nhe/Xho-rev	5´ - TTT CTC GAG GCT AGC GCT GCT GTT TCC GG
CH4436	Kpn-TEV-EC3006-ΔVENN-for	5´ - CTC GGT ACC GAG AAC CTG TAC TTC CAA TAT CTT AGC GTG TCT GAA AAG
CH4470	Kpn-TEV-3006-ΔVEN-for	5´ - CTG GGT ACC GAG AAC CTG TAC TTC CAA AAT TAT CTT AGC GTG TCT GAA
CH4471	Kpn-TEV-3006-ΔVE-for	5´ - CTG GGT ACC GAG AAC CTG TAC TTC CAA AAT AAT TAT CTT AGC GTG TCT GA
CH4932	Kpn-TEV-YmoI43969-ΔVENN-for	5´ - CTC GGT ACC GAG AAC CTG TAC TTC CAA AAC CTT AGT TTT GGC AAA GG
CH4933	YmoI43969-cdiI-Xho-rev	5´ - TTT CTC GAG TTA AGC AGG TAA TTT AGT CAG TAA AT
CH5112	3006-Kpn-TEV-Y5A-for	5´ - CTG GGT ACC GAG AAC CTG TAC TTC CAA GCT CTT AGC GTG TCT G
CH5183	EC3006-N4A-for	5´ - GCC GGT ACG GGG GCA CAG GCA GGT AGG AAC TCG GTT GAG AAT GCT TAT CTT AGC GTG TC
CH5184	EC3006-Y5A-for	5´ - GCC GGT ACG GGG GCA CAG GCA GGT AGG AAC TCG GTT GAG AAT AAT GCT CTT AGC GTG TC
CH5199	EC3006-S176-stop-Xho-rev	5´ - AAA CTC GAG CTA ACT ATT AGA CAA GCG TTG TTT AC
CH5298	EC3006-VENNN-for	5´ - GGT ACG GGG GCA CAG GCA GGT AGG AAC TCG GTT GAG AAT AAC AAT TAT CTT AGC GTG TCT
CH5299	EC3006-VENNL-for	5´ - GGT ACG GGG GCA CAG GCA GGT AGG AAC TCG GTT GAG AAT AAT CTT AGC GTG TCT GAA AA
CH5355	Yint-Kpn-TEV-ΔVENN-for	5´ - CTG GGT AAC GAG AAC CTG TAC TTC CAA TAT CTC AAT GCC AGT GAT AAG
CH5378	Yint-R181-stop-Xho-rev	5´ - GGT CTC GAG CTA CCG ATA GGG AAC CAA CTG AC
CH5467	TEV-ΔVEN-Yint-Kpn-for	5´ - TTT GGT ACC GAG AAC CTG TAC TTC CAA AAT CTC AAT GCC AGT GAT AA
CH5475	EC3006-Kpn-TEV-S174-for	5´ - TTT GGT ACC GAG AAC CTG TAC TTC CAA TCT AAT AGT TTC GAG GTT AGC TCA C
NB042	KpnI_6HTEV_3006F_dVENNY	5´ - CTC GGT ACC GAG AAC CTG TAC TTC CAA CTT AGC GTG TCT GAA AAG ACA
NB045	KpnI_6HTEV_3006F_dVENNYLSV	5´ - CTC GGT ACC GAG AAC CTG TAC TTC CAA TCT GAA AAG ACA GAG CTT GAG

[a] Restriction endonuclease sites underlined
