# Supplementary material for: Proteolytic processing induces a conformational switch required for antibacterial toxin delivery
Source: Nat Commun. 2022 Aug 29;13:5078. doi: 10.1038/s41467-022-32795-y (PMC9424206; doi:10.1038/s41467-022-32795-y)
Supplement: Supplementary file 3 — Reporting Summary [file 41467_2022_32795_MOESM3_ESM.pdf]

nature portfolio

Corresponding author(s): Christopher S. Hayes

Last updated by author(s): Aug 9, 2022

Reporting Summary

Nature Portfolio wishes to improve the reproducibility of the work that we publish. This form provides structure for consistency and transparency in reporting. For further information on Nature Portfolio policies, see our Editorial Policies and the Editorial Policy Checklist.

Statistics

For all statistical analyses, confirm that the following items are present in the figure legend, table legend, main text, or Methods section.

n/a	Confirmed	
☐	☒	The exact sample size (*n*) for each experimental group/condition, given as a discrete number and unit of measurement
☐	☒	A statement on whether measurements were taken from distinct samples or whether the same sample was measured repeatedly
☒	☐	The statistical test(s) used AND whether they are one- or two-sided<br>*Only common tests should be described solely by name; describe more complex techniques in the Methods section.*
☒	☐	A description of all covariates tested
☒	☐	A description of any assumptions or corrections, such as tests of normality and adjustment for multiple comparisons
☐	☒	A full description of the statistical parameters including central tendency (e.g. means) or other basic estimates (e.g. regression coefficient) AND variation (e.g. standard deviation) or associated estimates of uncertainty (e.g. confidence intervals)
☒	☐	For null hypothesis testing, the test statistic (e.g. *F*, *t*, *r*) with confidence intervals, effect sizes, degrees of freedom and *P* value noted<br>*Give P values as exact values whenever suitable.*
☒	☐	For Bayesian analysis, information on the choice of priors and Markov chain Monte Carlo settings
☒	☐	For hierarchical and complex designs, identification of the appropriate level for tests and full reporting of outcomes
☒	☐	Estimates of effect sizes (e.g. Cohen's *d*, Pearson's *r*), indicating how they were calculated

Our web collection on statistics for biologists contains articles on many of the points above.

Software and code

Policy information about availability of computer code

Data collection	HKL-3000 (release 708) obtained at http://www.hkl-xray.com/hkl-3000<br>CCP4 (version 7.0) obtained at https://www.ccp4.ac.uk/
Data analysis	Wolfram Mathematica (version 12.3); Wyatt ASTRA (version 8.1); Bio-Rad Quantity One (version 4.5); GraphPad Prism (version 9.4.0); Coot (version 0.8.2) https://www2.mrc-lmb.cam.ac.uk/personal/pemsley/coot/binaries/; PHENIX (version dev_2947) https://phenix-online.org/; NMRPipe (version 2012.090.12.09) and NMRDraw (version 8.2) https://www.ibbr.umd.edu/nmrpipe/; and UCSF Chimera (version 1.6), (https://www.cgl.ucsf.edu/chimera/).

For manuscripts utilizing custom algorithms or software that are central to the research but not yet described in published literature, software must be made available to editors and reviewers. We strongly encourage code deposition in a community repository (e.g. GitHub). See the Nature Portfolio guidelines for submitting code & software for further information.

Data

Policy information about availability of data

All manuscripts must include a data availability statement. This statement should provide the following information, where applicable:

- Accession codes, unique identifiers, or web links for publicly available datasets
- A description of any restrictions on data availability
- For clinical datasets or third party data, please ensure that the statement adheres to our policy

The data generated during this study are provided within the manuscript or the Supplementary Information files. Structure datasets are available in the Protein Data Bank under accession codes 6CP8 [http://doi.org/10.2210/pdb6CP8/pdb] and 6VEK [http://doi.org/10.2210/pdb6VEK/pdb]. Sequences for CdiAEC3006 and CdiIEC3006 are available from Genbank under accession codes EKI34460.1 [https://www.ncbi.nlm.nih.gov/protein/EKI34460.1/] and EKI34459.1 [https://www.ncbi.nlm.nih.gov/protein/EKI34459.1], respectively. Free induction decay (FID) NMR data have been deposited at the Biological Magnetic Resonance Data Bank [https://bmrb.io/] under accession number 51540.The source data underlying Figs. 1d-f, 3a-d, 4a-g, 5b-d, 6a-e, 7a-c, 8a-d and Supplementary Figs. 3, 4a-b, 5a-e, 6a-d, 7a-c, 7e-f, 9a-b, 10a-d are provided as Source Data files.

Human research participants

Policy information about studies involving human research participants and Sex and Gender in Research.

Reporting on sex and gender	not applicable
Population characteristics	not applicable
Recruitment	not applicable
Ethics oversight	not applicable

Note that full information on the approval of the study protocol must also be provided in the manuscript.

Field-specific reporting

Please select the one below that is the best fit for your research. If you are not sure, read the appropriate sections before making your selection.

☒ Life sciences ☐ Behavioural & social sciences ☐ Ecological, evolutionary & environmental sciences

For a reference copy of the document with all sections, see nature.com/documents/nr-reporting-summary-flat.pdf

Life sciences study design

All studies must disclose on these points even when the disclosure is negative.

Sample size	No statistical methods were used to determine sample sizes. Sample sizes were chosen according to accepted standards in the field and based on our recently published studies (PMCID:PMC6727969, PMCID:PMC6173971, PMCID:PMC6333426, PMCID:PMC5371414).
Data exclusions	No data were excluded.
Replication	All experiments in Figs. 3a, 6e, 7a, 8a and Supplementary Fig. 9a were repeated independently three times with similar results. Experiments depicted in Figs. 1d-f, 3b-d, 4a, 4c, 4e, 4g, 6a, 6c, 7b-c, 8b-d and Supplementary Figs. 4a, 9b and 10a were repeated independently at least twice with similar results. Experiments in Figs. 4b, 4d, 4f, 5b-d, 6b, 6d and Supplementary Figs. 3, 4b, 5a-d, 6a-e, 7a-d and 10b-d were performed once. For Figs. 7e-f, urea denaturation of CdiA-CTs was performed independently three times with similar results, and tRNase domain denaturation was performed once.<br>The HSQC experiments (Figs. 4b, 5c, 6b and Supplementary Figs. 6a-e, 7a-d) entail significant signal averaging from 2 to 12 h under carefully controlled conditions. In our experience, repetition does not result in meaningful variation in resonance position. This is illustrated by the overlay in Supplementary Fig. 6b, which shows the similarity between ΔVENN domain spectra acquired independently at 20 µM and 230 µM.<br><br>Similar to the NMR experiments, circular dichroism (CD) spectroscopy is very reproducible with highly purified and accurately quantified protein samples as illustrated by the reproducibility of CD spectra in Figs. 4a and 6a. The experiments in Figs. 4d and 5b were only performed once because the CD spectra of ΔVENN-Y5A and ΔVEN entry domains closely overlap the spectra of the corresponding wild-type ΔVENN domains as expected for nearly identical proteins.<br><br>Although outer-membrane bypass with ΔVENN-Y5A was performed only once (Fig. 4f), the ΔVENN CdiA-CT shown in this experiment yielded the same results in six other independent replicates as illustrated in Figs. 1d, 1f and 6e. All other CdiA-CT variants were also replicated with similar results. Thus, the outer-membrane bypass assay is reproducible between independent experiments.<br><br>The urea melts in Fig. 5d were only performed once because the quantitative difference in thermodynamic stability is unlikely to occur by chance or through experimental error. We also note that other chemical melts shown in Figs. 4e, 6c and Supplemental Fig. 7e (CdiA-CT) were

reproducible upon independent repetition. For these reasons, we also performed the tRNase domain melt in Supplementary Fig. 7e only once.

The in vitro nuclease assay in Fig. 6d was performed only once because this assay is highly reproducible as illustrated by the independent experiments shown in Figs. 1e and 4g. Moreover, we have previously performed and published in vitro nuclease assays with this same toxin in PMCID: PMC6834915.

The experiment in Supplementary Fig. 3 was performed only once because this is a standard SDS-PAGE analysis. Though not run on the same gel as a true replicate, all protein samples used in this study were similarly analyzed to monitor purity.

The experiment in Supplementary Fig. 4b was performed only once because the data suggest that there is no significance difference in dye accessibility between ΔVEN and ΔVENN entry domains.

Although the presented size-exclusion chromatography experiments in Supplemental Figs. 5a-e were each performed once, we have analyzed these protein preps by size-exclusion using other HPLC and FPLC instruments. All three chromatography experiments yield the same relative elution profiles. The experiment in Supplemental Fig. 5d also provides information about the reproducibility of multi-angle light scattering data.

The HPLC and mass spectrometry analysis in Supplemental Figs. 10b-c identified the cleavage site unequivocally. Given that the vesicle cleavage assay in Supplemental Fig. 10a yielded the same results upon replication, we presume that the same peptide bond was cleaved in both experiments. Though the experiment in Supplemental Fig. 10d was only performed once as shown, we performed additional time-course experiments to determine the 40 min end-point for the presented figure.

Randomization

Randomization was not required for this study because for each experiment, all samples were simultaneously treated and analyzed in parallel.

Blinding

All results are based on objective and/or quantitative analyses of primary capture data, with no subjective interpretations. Therefore, blinding was not required for this study.

Reporting for specific materials, systems and methods

We require information from authors about some types of materials, experimental systems and methods used in many studies. Here, indicate whether each material, system or method listed is relevant to your study. If you are not sure if a list item applies to your research, read the appropriate section before selecting a response.

Materials & experimental systems

n/a	Involved in the study
☐	☒ Antibodies
☒	☐ Eukaryotic cell lines
☒	☐ Palaeontology and archaeology
☒	☐ Animals and other organisms
☒	☐ Clinical data
☒	☐ Dual use research of concern

Methods

n/a	Involved in the study
☒	☐ ChIP-seq
☒	☐ Flow cytometry
☒	☐ MRI-based neuroimaging

Antibodies

Antibodies used

Custom rabbit polyclonal antisera (raised against residues Val33-Gly285 of CdiA) was used as the primary antibody at 1:10,000 dilution, and IRDye 800CW goat anti-rabbit IgG (LI-COR, Cat# P/N 925-32211) served as the secondary antibody used at 1:40,000 dilution.

Validation

The anti-CdiA polyclonal antibody was validated using whole-cell lysates from Escherichia coli cells that do not produce CdiA. These validation data are shown in Figure 7 from Ruhe et al. (2015) Molecular Microbiology 98:175-93, which is reference [67] in the manuscript. Additional validation with other CdiA deletion constructs has been published in Ruhe et al. (2018) Cell 175:921-33, which is reference [2] in the manuscript.
